# Labeling and Tracking of Individual Human Mesenchymal Stromal Cells Using Photoconvertible Fluorescent Microcapsules

**DOI:** 10.3390/ijms241713665

**Published:** 2023-09-04

**Authors:** Olga A. Sindeeva, Polina A. Demina, Zhanna V. Kozyreva, Albert R. Muslimov, Olga I. Gusliakova, Valeriia O. Laushkina, Ekaterina A. Mordovina, Daria Tsyupka, Olga S. Epifanovskaya, Anastasiia Yu. Sapach, Irina Yu. Goryacheva, Gleb B. Sukhorukov

**Affiliations:** 1 Vladimir Zelman Center for Neurobiology and Brain Rehabilitation, Skolkovo Institute of Science and Technology, Skolkovo Institute of Science and Technology, Bolshoy Boulevard 30, 121205 Moscow, Russia; zhanna.kozyreva@skoltech.ru (Z.V.K.); olga.gusliakova17@gmail.com (O.I.G.); anastasiia.sapach@skoltech.ru (A.Y.S.); goryachevaiy@mail.ru (I.Y.G.); 2 Science Medical Center, Saratov State University, 83 Astrakhanskaya Str., 410012 Saratov, Russia; polina.a.demina@list.ru (P.A.D.); mordovina_ekaterina@mail.ru (E.A.M.); tsyupkadv@mail.ru (D.T.); 3 Scientific Center for Translational Medicine, Sirius University of Science and Technology, 1 Olympic Ave., 354340 Sirius, Russia; albert.r.muslimov@gmail.com; 4 Laboratory of Nano and Microencapsulation of Biologically Active Substances, Peter the Great St. Petersburg Polytechnic University, Polytechnicheskaya 29, 195251 St. Petersburg, Russia; 5 RM Gorbacheva Research Institute, Pavlov University, L’va Tolstogo 6-8, 197022 St. Petersburg, Russia; valeriia.laushkina@gmail.com (V.O.L.); epif-olga@rambler.ru (O.S.E.); 6 School of Engineering and Materials Science, Queen Mary University of London, Mile End Road, London E1 4NS, UK

**Keywords:** human mesenchymal stromal cells, cell imaging, cell tracking, cell migration, photoconversion label, fluorescent label, Rhodamine B, hydrothermal encapsulation, thermally treated microcapsules

## Abstract

The behavior and migration of human mesenchymal stromal cells (hMSCs) are focal points of research in the biomedical field. One of the major aspects is potential therapy using hMCS, but at present, the safety of their use is still controversial owing to limited data on changes that occur with hMSCs in the long term. Fluorescent photoconvertible proteins are intensively used today as “gold standard” to mark the individual cells and study single-cell interactions, migration processes, and the formation of pure lines. A crucial disadvantage of this method is the need for genetic modification of the primary culture, which casts doubt on the possibility of exploring the resulting clones in personalized medicine. Here we present a new approach for labeling and tracking hMSCs without genetic modification based on the application of cell-internalizable photoconvertible polyelectrolyte microcapsules (size: 2.6 ± 0.5 μm). These capsules were loaded with rhodamine B, and after thermal treatment, exhibited fluorescent photoconversion properties. Photoconvertible capsules demonstrated low cytotoxicity, did not affect the immunophenotype of the hMSCs, and maintained a high level of fluorescent signal for at least seven days. The developed approach was tested for cell tracking for four days and made it possible to trace the destiny of daughter cells without the need for additional labeling.

## 1. Introduction

Human mesenchymal stromal cells (hMSCs) are at the center of the biomedical research community because of their unique properties, namely: multipotency [1,2,3,4], self-renewal capacity [5], long-term ex vivo proliferation capacity [6,7], ability to migrate to tissue injury areas [8,9], and immune modulation [10]. These cells can also secrete anti-inflammatory cytokines [11] and hide themselves from the immune system [12]. hMSCs have great potential in target [13,14] and regenerative [15] medicine and have already been applied in clinical practice [16]. The ability of hMSCs to differentiate into multiple lineages opens up broad prospects for tissue engineering [7]. Nevertheless, the possibility of spontaneous differentiation (e.g., by cancer cells or fibroblasts [17,18]) raises concerns. At the same time, the ability of the cells to migrate in a directed manner and carry cargo to a site of damage has wide potential [19]. In this regard, the study of the fundamental issues of hMSCs interaction with healthy and pathological cells and the processes of their migration and differentiation remain extremely relevant.

Today, one of the key approaches for studying the behavior and properties of individual cells in heterogeneous populations is the use of photoconvertible GFP-like proteins (mEosFP, tdEosFP, Dendra, Dronpa, Kaede, KikGR, mOrange, etc.) [20,21,22,23,24,25]. This technique makes it possible to mark a defined cell or cell ensemble using localized laser exposure. Since the effect of protein photoconversion is irreversible, researchers are able to track the changes that occur in selected cells over time [26,27]. This approach offers the possibility of not only tracing the destiny of individual cells in heterogeneous populations but also of marking and re-sorting cells with certain requested characteristics for cultivation and growth (e.g., creation of purified cell lines) [28]. However, this approach has its limitations: (i) some GFP-like proteins exhibit toxicity [29] and a tendency to aggregate [30], (ii) a gradual decrease of the fluorescent signal during cell doubling (i.e., daughter cells share a part of the converted protein among themselves until the signal disappears completely) [31], (iii) the inability to identify an individual cell among the marked ones, and (iv) the need for gene modification of the primary culture carrying the gene of the photoconvertible protein [32]. Gene modification is the main limitation to exploiting the resulting cell clones in personalized medicine because of the probability of random mutations occurring during transfection.

In our previous study, a promising new highly stable fluorescent photoconversion label was presented. This label is a thermally treated polymer multilayer microcapsule loaded with the fluorescent dye Rhodamine B (RhB), which can be easily internalized by various cell lines and reliably detected in the cytoplasm [33,34]. Cells are capable of internalizing several microcapsules, which makes it possible to combine photoconverted and unconverted labels inside one cell and create color coding. This allows us to identify a specific cell among several marked cells. In addition, this method does not require genetic modification of the cell line.

It is important to note that the efficiency of internalization and the effect on metabolic activity differed among all previously studied cell lines such as cancer cells (HeLa and B16F10); mouse monocyte macrophage cell lines (Raw 264.7); mouse myoblasts (C2C12); mouse fibroblasts (L929); and human embryonic fibroblasts (ChEF 392/1)) [33]. This requires careful selection of the labeling protocol for each specific culture. In addition, the effect of microcapsules on cells over time and the possibility of long-term tracking (several days) have not been previously studied.

In this study, we explored and tested new fluorescent photoconvertible microcapsules for the prolonged labeling and tracking of individual bone marrow hMSCs in primary cell culture. Here, the influence of capsule number on viability, the efficiency of internalization, and the effect on the phenotype during long-term incubation (1 week) were studied. hMSCs tracking over seven days is also presented and analyzed.

## 2. Results and Discussion

### 2.1. Synthesis and Characterization of Photoconvertible Microcapsules with Rhodamine B

**Figure 1:**
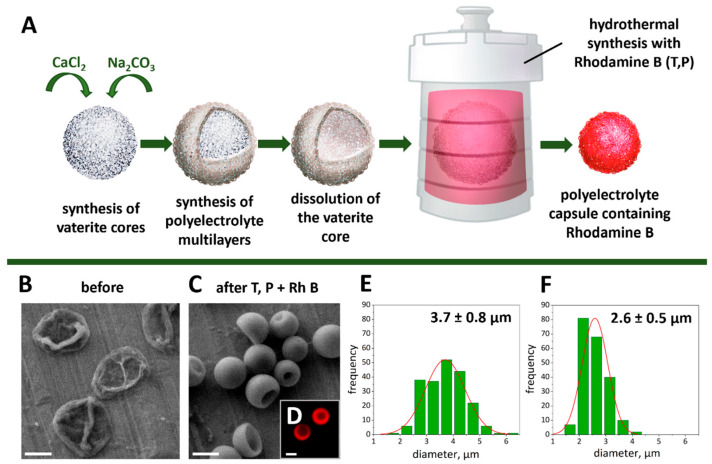
Scheme of Rhodamine B encapsulation in polyelectrolyte microcapsules (**A**). Typical SEM images of microcapsules before (**B**) and after (**C**) the hydrothermal synthesis. CLSM image of typical thermal treated polyelectrolyte microcapsules with Rhodamine B (**D**). Size distribution with Gaussian fit of microcapsules before (**E**) and after (**F**) hydrothermal synthesis in an aqueous solution. The diameter was determined from the SEM images of 200 microcapsules. The scale bar is 2 µm.

To implement the possibility of labeling and tracking hMSCs, thermally treated polyelectrolyte microcapsules containing RhB were synthesized according to the method described in our previous work [33]. The main scheme of RhB encapsulation into the polyelectrolyte microcapsules is shown in Figure 1A. In the first stage of the study, polymer multilayers based on poly(allylamine hydrochloride) and poly(4-styrene sulfonate) sodium salt (PAH/PSS) were successively formed on the vaterite core templates. The microcapsules were incubated in dextran sulfate sodium salt (DS) aqueous solution after core dissolution. Such procedures provide an additional source of carbon structure in the capsule composition, which is formed from polymers during hydrothermal synthesis [35]. DS-based carbon structures have been demonstrated to exhibit significant photocatalytic activity [33], which is important for the photocatalytic conversion of RhB.

Hydrothermal synthesis in the presence of RhB led to a significant change in the shape (Figure 1B,C) and decrease in the size (Figure 1E,F) of the initial microcapsules. The size decrease is associated with the compression of polyelectrolyte shells under high-temperature conditions [36] Due to heat treatment (180 °C, 3 h), the capsule size decreased from the initial 3.7 ± 0.8 to 2.6 ± 0.5 μm. At the same time, the fluorescent dye remained securely encapsulated inside the microcapsule, as shown in Figure 1D, which is consistent with data obtained by other authors [37]. Electrokinetic ζ-potential before and after hydrothermal synthesis with RhB was −15.8 ± 0.5 mV and −20.7 ± 2.2 mV, respectively.

### 2.2. Thermally Treated Polyelectrolyte Microcapsules Fluorescence Conversion under 561 nm Laser Irradiation in Aqueous Medium and the Microenvironment of hMSCs

Since the photoconversion of RhB-based labels depends on different conditions such as irradiation wavelength, laser power density, etc., [33,34] for a successful photoconversion procedure it is necessary to select parameters with minimal impact on the system as a whole.

**Figure 2:**
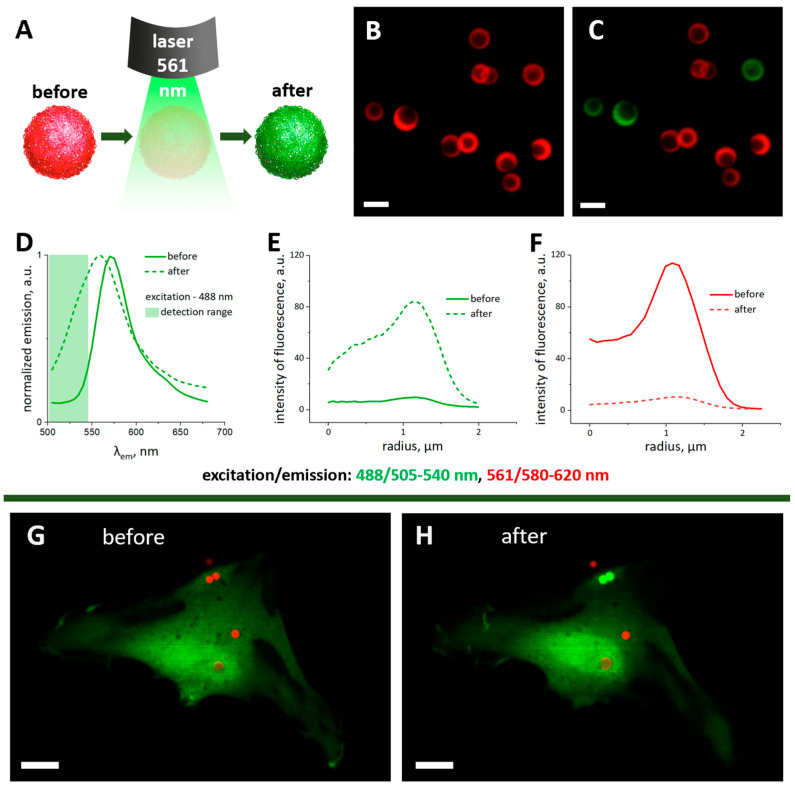
Scheme of photoconversion of thermally treated polyelectrolyte microcapsules with Rhodamine B under 561 nm laser irradiation (**A**). CLSM images of microcapsules before (**B**,**C**) and after 561 nm laser irradiation. The scale bar is 2 µm. Microcapsule emission spectra under 488 nm excitation before and after laser irradiation (green rectangle corresponds to the 505−540 nm detection range) (**D**). Angularly averaged fluorescence intensity of microcapsules in the 500−545 (**E**) and 560−650 nm (**F**) detection ranges (excitation—488 and 561 nm, respectively) before and after 561 nm laser irradiation. CLSM images of microcapsules before (**G**) and 20 min after (**H**) laser irradiation on hMSCs (microcapsules before (red) and after (green) photoconversion, cytoplasm stained with vital dye—Calcein AM (green)). The scale bar is 10 µm.

A continuous diode laser with a power of 50 mW and wavelength of 561 nm was used to obtain successful capsule photoconversion (Figure 2A). A photoconversion scanning area of 12.11 × 12.11 μm was chosen to maximize laser irradiation for other microcapsules and exclude their accidental photoconversion. Thermally treated polyelectrolyte microcapsules demonstrated bright fluorescence in the emission range of 580–620 nm (Figure 2B, red color) before photoconversion using 561 nm laser irradiation. After photoconversion, a bright fluorescence signal appeared in the range of 505–540 nm (Figure 2C, green color). The emission spectra (so-called lambda-scans) obtained before and after the 561 nm photoconversion procedure at an excitation wavelength of 488 nm demonstrated a hypsochromic shift of the emission spectrum maximum (Figure 2D) and an increase of fluorescence signal in the detection range of 505–540 nm (Figure 2D, light green rectangle). Spectral changes were confirmed by analyzing the capsule’s average fluorescence intensities before and after 561 nm irradiation. Fluorescence intensity increased in the 505–540 nm emission range (Figure 2E) and decreased in the 560–650 nm emission range (Figure 2F).

The next step was to confirm that a 561 nm photoconversion procedure was suitable for individual cell tracking in the case of the hMSCs cell line and did not cause cell death. The vital dye Calcein AM was used to control the survivability of cells after photoconversion. A single hMSCs cell with a few internalized thermally treated microcapsules (Figure 2G) was selected. After photoconversion, the two chosen microcapsules acquired bright green fluorescence and the cell remained alive (Figure 2H).

Note that in our experiments, we used a power density of 451 kV/cm^2^ during the photoconversion procedure, but the beam area was only 0.776 µm^2^. The selected parameters used for photoconversion did not cause cell death (Figure 2J,H). Similar results were obtained in our previous study of different cell types [33,34]. In addition, during photoconversion, we set the minimum possible scanning area and mainly acted on the part of the cell containing the capsule. This minimized the impact of the laser on cell membranes and organelles.

In our first study, it was shown that the efficiency of microcapsule photoconversion depends significantly on the wavelength, and the optimum wavelength is 554 nm (absorption peak of RhB) [33]. Nevertheless, not all optical systems are equipped with this type of laser. Therefore, in the next study, we evaluated the possibility of the photoconversion of microcapsules using the most common constant and pulsed lasers at 532 nm [34]. Here, we used a standard confocal laser with a wavelength of 561 nm for the photoconversion. Taken together, our data show that the developed fluorescent labels can be photoconverted using various types of lasers (at least in the range of 532–561 nm). This makes the developed approach useful to a wide range of researchers. Note, the photoconversion settings must be optimized for a particular optical system.

### 2.3. Influence of the Microcapsules’ Quantity on Cell Viability and Internalization

Cell internalization and viability studies were conducted to evaluate the effectiveness of hMSCs labeling using thermally treated polyelectrolyte microcapsules loaded with RhB. The MTT assay was used to test cell viability. This assay allows for the indirect evaluation of the relative cytotoxicity of objects or substances based on changes in cell metabolic activity.

**Figure 3:**
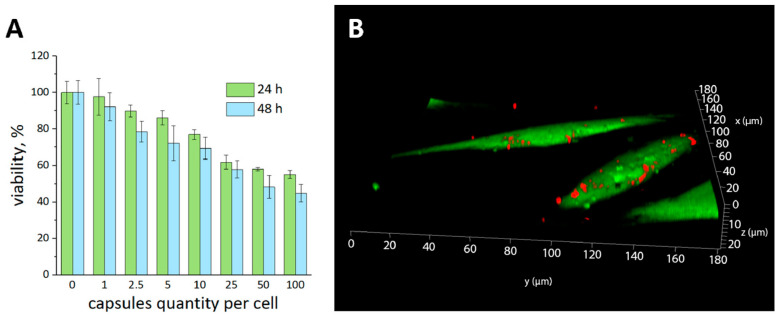
Viability dependence of hMSCs on the number of added thermally treated polyelectrolyte microcapsules (**A**). 3D reconstruction of hMSCs 24 h after incubation with microcapsules (microcapsules (red), cytoplasm stained with vital dye—Calcein AM (green)) (**B**).

**Figure 4:**
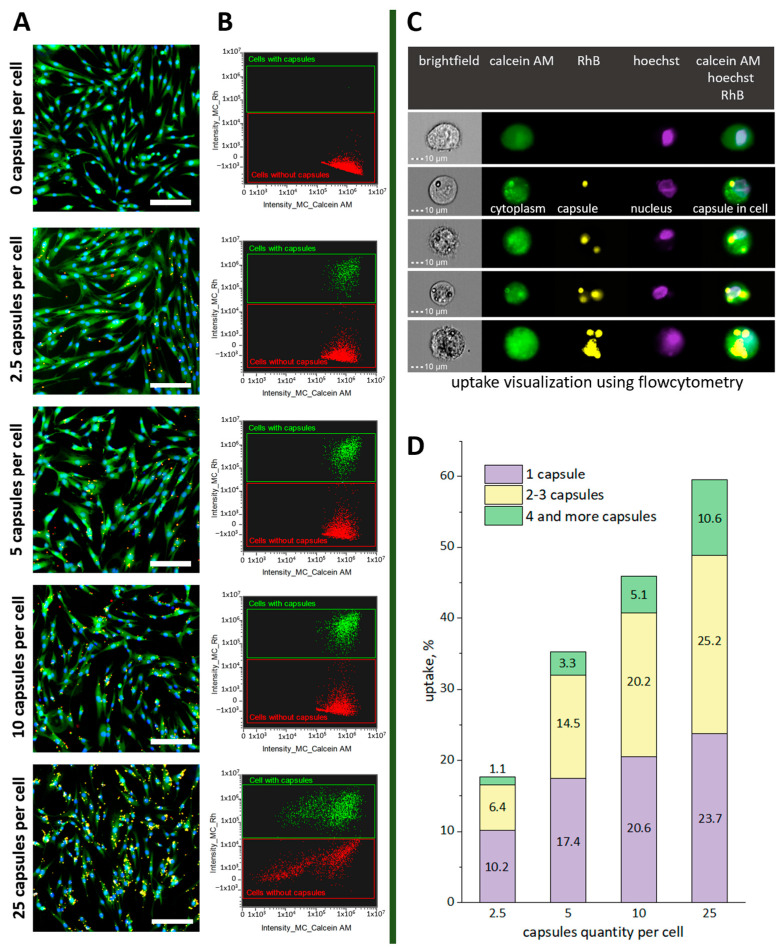
Fluorescence images of hMSCs 24 h after incubation with different concentrations of microcapsules (cytoplasm stained with the vital dye—Calcein AM (green), cell nucleus—Hoechst 33258 (blue), and microcapsules—Rhodamine B (red)). The scale bar is 200 µm (**A**). Cytograms of Calcein AM and RhB fluorescence intensity visualizing the subpopulations of cells (Calcein AM) associated with fluorescence specific for capsules (RhB) depending on the concentration of the added capsules (**B**). Optical and fluorescent images of hMSCs were obtained using a flow cytometer with an object imaging system (**C**). Dependence of the percentage of cells in the population that internalized the capsules on the concentration of the added capsules (**D**).

The hMSCs’ viability was studied after 24 and 48 h of co-incubation at different microcapsule concentrations (ratios: 0, 1, 2.5, 5, 10, 25, 50, and 100 capsules per cell). The test showed a gradual decrease in the hMSCs’ viability with an increase in the number of microcapsules, which became critical at a ratio of 25 capsules per cell (Figure 3A). At the same time, the 3D reconstruction showed that 24 h after cultivation, a significant portion of the capsules was associated with the cell membrane but was not located inside the cells (Figure 3B). According to this, a more detailed study of the microcapsules internalization was performed using a cytometer with an object imaging system (Figure 4).

For this, the cells were incubated with capsules in the wells of a 24-well plate at ratios of 2.5, 5, 10, and 25 capsules per cell. Next, the cytoplasm was stained with a vital dye—Calcein AM—and the nucleus was stained with Hoechst. Visual control of cell staining before trypsin treatment and measurement using a flow cytometer showed a significant change in cell morphology at a ratio of 25 capsules per cell (Figure 4A).

Cell flow cytometry results after 24 h of incubation with the capsules clearly separated the proportion of cells that captured the capsules (Figure 4B). The internalization efficiency for the ratio of 2.5, 5, 10, and 25 capsules per cell was 17.7 ± 3.4, 35.3 ± 3.2, 46 ± 2, and 60 ± 4.7%, respectively. The main trend was an increase in the number of both the total cells that internalized capsules and of each individual group considered (1, 2–3, 4, or more capsules per cell) (Figure 4C,D). In addition, the number of cells that captured a larger number of capsules increased with an increase in the concentration of added capsules. For instance, when 25 capsules per cell were added, the percentage of cells with 4 capsules or more was an order of magnitude greater than in samples where 2.5 capsules per cell were added. The proportion of the subpopulation with 2–3 capsules per cell increased by four times while the proportion of cells that captured only one capsule increased by only two times.

Notably, a slightly different presentation may appear visually during sample microscopy, with a tendency to increase the total number of cells associated with capsules. Using cytometry it is possible to determine the rate of cells tightly associated with capsules, even if they are still located on the cell membranes. Only internalized capsules or ones tightly bound to the membrane can withstand detachment procedures and several washes with DPBS. The ability to view fluorescent images of all objects identified with the cytometer significantly improved the selectivity and quality of the obtained data.

In general, a direct relationship was observed between cell viability and the number of captured capsules. The optimal ratio was 10 capsules per cell as an added suspension, which provided sufficient hMSCs labeling at an acceptable viability rate. Other authors also did not observe acute toxicity at moderate capsule concentrations (the outermost polyanionic layer appeared to further decrease toxicity) [38]. Some toxicity was observed at elevated capsule concentrations, which may be associated with capsule sedimentation on the cells’ surface as a result of competition for space between capsules and cells. This effect decreases the metabolic activity of cells, thus affecting their viability [39,40,41]. Our previous studies on various cell lines showed that metabolic activity after cultivation with photoconversion microcapsules varied for different cell lines, which is also associated with the number of internalized microcapsules [33].

It has been reported that polymeric particles are poorly internalized by suspension cell culture (such as Jurkat) in, and predominantly located in clusters on, the cell surface [42]. This may become a limitation for labeling suspension cultures with photoconvertible microcapsules.

### 2.4. Stability Evaluation of the hMSCs’ Immunophenotype during Co-Incubation with Thermally Treated Polyelectrolyte Microcapsules

hMSCs are non-hematopoietic cells that may be obtained from various tissues and organs, including the bone marrow, Wharton’s jelly, adipose tissue, peripheral blood, umbilical cord, amniotic fluid, placenta, and dental pulp [43,44,45]. The common characterization markers of hMSCs are CD105, CD90, and lack the expression of CD45 and CD34 [46,47]. Note, hMSCs can undergo not only morphological but also phenotypic changes under the influence of cultivation conditions (long-term cultivation, medium composition, etc.) [48]. Therefore, the constant monitoring of morphology and phenotype retention is a key factor in conducting reliable studies on hMSCs.

**Figure A1:**
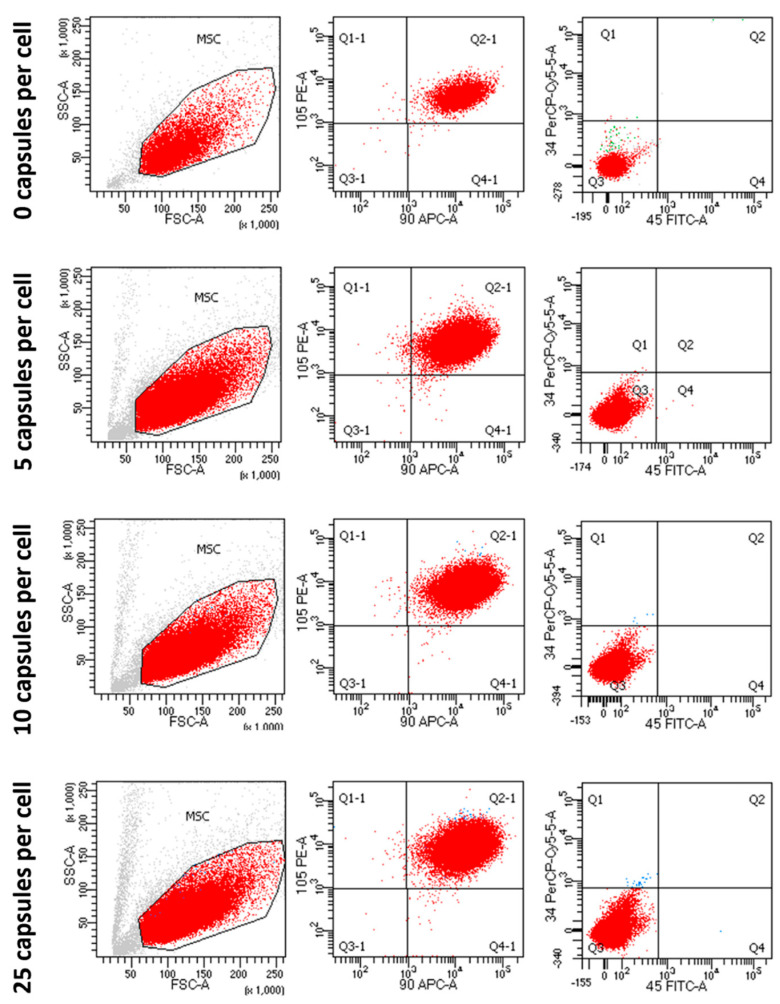
Representative bivariate dot plots illustrating the gating strategy and immunophenotypic characteristics of bone marrow CD45-, CD34-, CD105+, and CD90+ hMSCs incubated with thermally treated polyelectrolyte microcapsules for 1 day.

**Figure A2:**
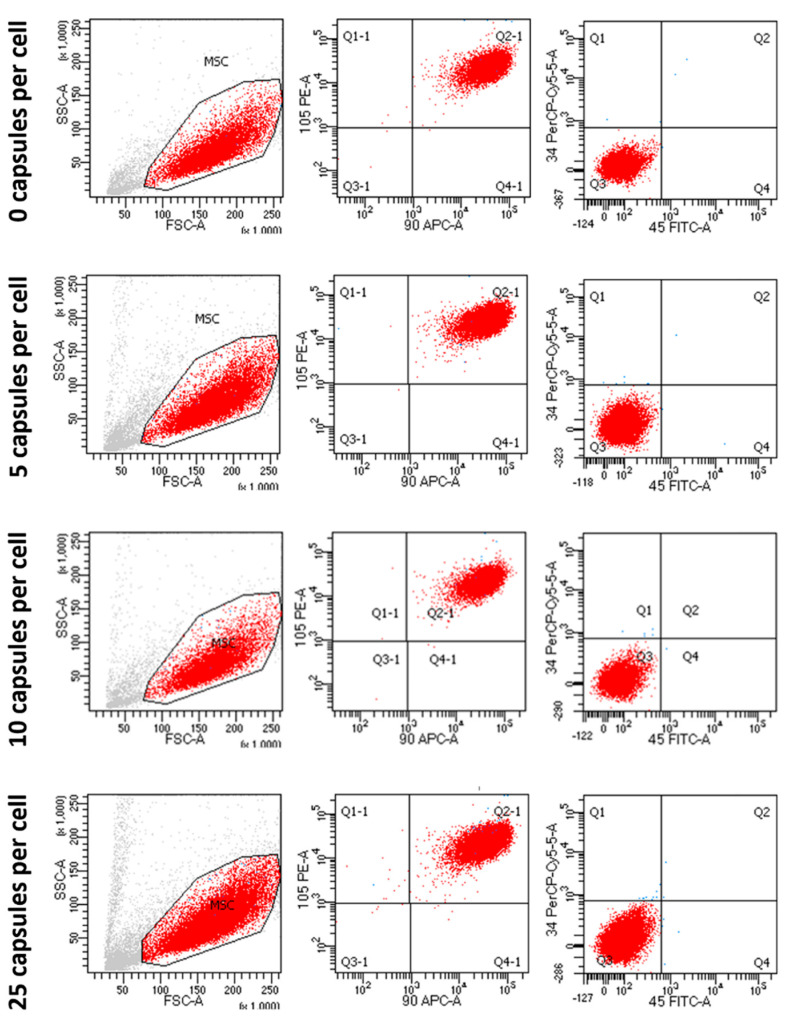
Representative bivariate dot plots illustrating the gating strategy and immunophenotypic characteristics of bone marrow CD45-, CD34-, CD105+, and CD90+ hMSCs incubated with thermally treated polyelectrolyte microcapsules for 7 days.

Therefore, the effect of microcapsules on the hMSCs’ phenotype was evaluated in the next step. At all the studied microcapsule-to-cell ratios (5, 10, and 25 capsules per cell) no effect on the cell phenotype was observed. On the first day (Figure A1), as well as on the seventh day (Figure A2) of incubation, the phenotype of the cells did not differ from that of the control sample (CD34, CD45—less than 1% of the population; CD90, CD105—more than 99% of the population).

In general, no change in the hMSCs’ immunophenotype during long-term cultivation (7 days) with photoconvertible microcapsules is extremely important to establish for the correct interpretation of the obtained experimental data.

### 2.5. hMSCs Tracking

**Figure 5:**
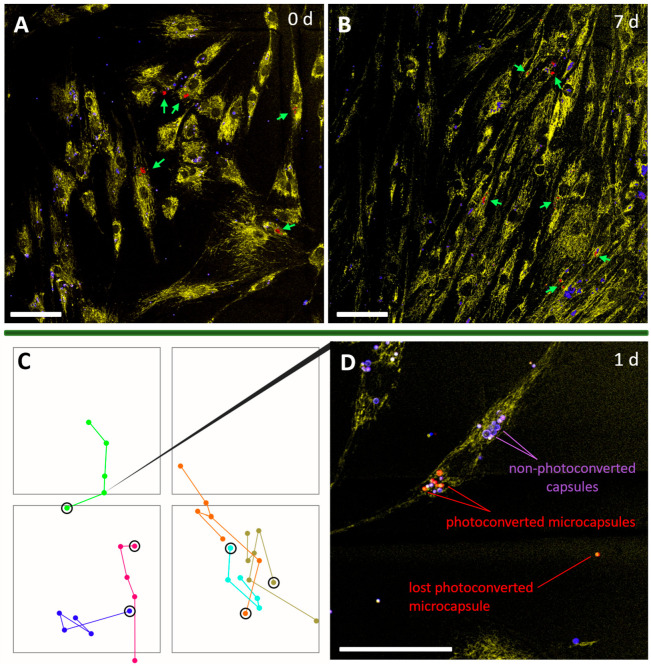
CLSM images of hMSCs with microcapsules at 0 (**A**) and 7 (**B**) days after labeling (hMSCs—yellow (dye—Cy7), non-photoconverted microcapsules—blue, photoconverted—red). Green arrows indicate the photoconverted microcapsules. The scale bar is 100 µm. (**C**) Tracking diagram of six cells over 4 days. The dots circled in black indicate day 0. The remaining dots show the position of the nucleus of the labeled cell every 24 h. The side of each square is 500 μm. Tracks of individual cell movement are marked with different colors. (**D**) Image of one of the marked cells 1 day after the tracking commenced. The scale bar is 50 µm.

Finally, the possibility of using photoconvertible capsules as a label for prolonged (several days) tracking of hMSCs was evaluated. First, it was shown that converted capsules can be reliably detected in cells for at least one week after labeling (Figure 5A,B). Most likely, the capsules will retain a bright fluorescent signal for a long time (several weeks or months) because their composition is based on non-biodegradable polyelectrolytes [49]. Nevertheless, the duration of hMSCs tracking is largely limited by the rapid proliferation of MSCs.

Rapid hMSCs proliferation was also noted in this experiment, which is consistent with the data reported by other authors. The doubling period varies from 14 to 68 h for different types of stem cells [50,51,52,53]. The average doubling period for bone marrow hMSCs is 49.9 ± 4.2 h [52]. After approximately 7 days, the cell culture reached confluence when the initial planting density was 25–30% (Figure 5B). Thus, observation for more than 4–5 days does not make sense for bone marrow hMSCs.

To illustrate the applicability of the method, six cells were tracked in four squares of a Petri dish (Figure 5C; the side of each square corresponded to 500 μm). Cell images were obtained every 24 h for 4 days. Each of the marked cells was characterized by its unique trajectory of movement. During the observation period, the division of two marked cells occurred, while the daughter cells were also well identified (tracks marked in orange and dark green) owing to the preservation of a bright fluorescent signal from the capsules.

Some of the photoconverted microcapsules may have been lost by cells during the tracking process. This was probably due to their localization on the membrane and incomplete internalization by the cell at the time of marking (Figure 5B).

Generally, these data show that reliable detection requires the presence of several photoconverted capsules inside the cell, particularly after doubling. Tracking also requires periodic imaging of the cells under study to identify the new coding of daughter cells resulting from doubling (random microcapsule splitting between daughter cells).

hMSCs are extremely sensitive to their microenvironment and intercellular contacts. The optimal conditions for their cultivation are confluency in the range of 50–80% when they exhibit high metabolic activity as stated in [53]. Low and high levels of confluence (10–20% and 100%, respectively) inhibit the growth and proliferation of the culture and can also significantly affect the properties of hMSCs and even start the process of differentiation. That is what we have to avoid in our study. We started experiments with approximately 25–30% confluency (1.5 × 10^4^/cm^2^ cells), which is optimal for efficient labeling (Figure 4) and is consistent with standard culture protocols for this cell type. Thus, the practical limitation was about 1.5–2 doubling periods before reaching 100% confluency by day 7 (Figure 5B). During this period, the photoconverted capsules retained a bright fluorescent signal and the capsule number split between daughter cells still kept them traceable. At the same time, it is statistically possible that subsequent doubling might produce daughter cells eventually without a single capsule. This may be a limitation of the technique in the case of labeling cell lines with less sensitivity to the microenvironment and allowing cultivation at a lower level of confluence. This problem might be overcome with a higher initial capsule-to-cell ratio, although it might still persist in some cases.

Nevertheless, as we compare our approach with photoconvertible proteins the latter also have limitations. In their case, the label is not “split” but “diluted” due to two parallel processes (physical splitting of the converted protein molecules between daughter cells and dilution of the converted molecules by constantly synthesized unconverted ones) [31]. This leads to a significant decrease in the fluorescence brightness of the protein label during cell proliferation, which can be critical even 48–72 h after photoconversion and requires additional labeling. In the case of photoconvertible capsules, the brightness of single capsule remains as it was and remains visible, but the number of capsules per cell during doubling and the “color code” change can be still traceable with less options for “color code”. This is an advantage in certain cases.

The detection and tracking of hMSCs labeled with photoconvertible capsules in animal models is still a difficult task. The penetration of laser irradiation into biological tissues is usually limited to approximately 50–70 μm [54]. This is due to light scattering caused by refractive index (RI) mismatches at the interfaces between biological tissue components such as lipids, proteins, and water [55]. Optical clearing agents may be key in solving this problem in the future [56]. We believe that this approach will significantly expand the range of applications of the developed photoconvertible labels. It has been shown that microcapsules based on synthetic polyelectrolytes (PAH/PSS) can remain in cells and be reliably detected for more than 30 days [57]. This opens up broad prospects for their use as a label for long-term cell tracking in vivo and in vitro.

## 3. Materials and Methods

### 3.1. Materials

Poly(4-styrene sulfonate) sodium salt (PSS, Mw = 70 kDa), poly(allylamine hydrochloride) (PAH, Mw = 17.5 kDa), dextran sulfate sodium salt (DS, Mw = 40 kDa), ethylenediaminetetraacetic acid disodium salt, calcium chloride, sodium carbonate, and Rhodamine B were purchased from Sigma-Aldrich (Taufkirchen, Germany). Dimethyl sulfoxide was purchased from Merck (Darmstadt, Germany). Cyanine 7 NHS ester (Cy7) was obtained from Lumiprobe (Moscow, Russia). Polyvinyl alcohol (PVA) powder (Mw = 72,000 g/mol with a degree of hydrolysis of 85–89%) was supplied by AppliChem GmbH (Darmstadt, Germany). All the chemicals were used without further purification. Deionized water produced using a Milli-Q Plus 185 water treatment system (Merck Millipore, Darmstadt, Germany) was used to prepare all solutions. Phosphate-buffered saline (PBS), Calcein AM fluorescent dye, and the MTT cell proliferation assay kit were purchased from Thermo Fisher Scientific (Eugene, OR, USA).

### 3.2. Instruments

Milli-Q Plus185 (Millipore, Darmstadt, Germany), ClarioSTAR Plus (BMG Labtech, Ortenberg, Germany), Scanning electron microscope VEGA III (TESCAN, Brno, Czech Republic), Zetasizer Ultra Red Label (Malvern Panalytical, Malvern, UK), Leica TCS SP8X CLSM (Leica Biosystems, Wetzlar, Germany), ImageStream X Mark II Imaging flow cytometer (Luminex, Austin, TX, USA), and BD FACSCanto™ Clinical Flow Cytometry System (BD Biosciences, San Jose, CA, USA).

### 3.3. Synthesis and Characterization of Photoconvertible Microcapsules with Rhodamine B

The photoconvertible capsules were prepared according to the procedure described by Demina et al. [33] with some modifications. First, to synthesize vaterite cores, equal volumes (0.650 μL) of 1 M calcium chloride dihydrate and 1 M sodium carbonate were combined by constant mixing in 3 mL of water. The resulting particles were washed three times with water and then alternating polyelectrolyte layers (PSS and PAH, 1 mg mL^−1^ in 0.15 M NaCl) were applied. For the adsorption of each layer, the sample was placed on the rotator for 10 min and then washed three times with deionized water (DI) by centrifugation (2 min, 1400 g). After 4 PAH/PSS bilayers were obtained, EDTA 0.2 M aqueous solution was used to dissolve the inner vaterite cores. Finally, the DS aqueous solution (2 mg mL^−1^) was added to the sample, which was left on the rotator for 1 h followed by washing three times using centrifugation. Then, 0.5 mL of Rhodamine B solution (0.5 mg/mL) was added to the capsules and thoroughly mixed in 17% PVA water gel. The prepared sample was placed in a high-pressure autoclave for hydrothermal treatment (180 °C, 3 h). After cooling, the capsules were washed in DI water several times until the PVA gel was completely removed (5600 g × 4 min).

Scanning electron microscopy (SEM) images of the capsules were obtained using a VEGA 3 LM, TESCAN (Czech Republic, Brno). The samples were prepared by depositing a drop of the capsule suspension on copper tape and leaving it to dry at room temperature. The samples were coated with a thin gold film (approximately 5 nm thick) using a Q150R ES Plus rotary-pumped coater (Quorum, Laughton, England). SEM images analysis was performed using the ImageJ software 1.54f.

### 3.4. Fluorescence Imaging and Photoconversion Technique

Confocal laser scanning microscopy (CLSM) was performed using a confocal laser scanning microscope Leica TCS SP8 X (Leica, Wetzlar, Germany) equipped with an immersion objective of 20×\0.70 NA. For microcapsule exposure and excitation, several discrete lasers were used (488 and 561 nm). RhB fluorescence was excited at 561 nm and the emission range was 580–620 nm (red channel). Calcein AM and photoconverted microcapsules fluorescence was excited at 488 nm and the emission range was 505–540 nm (green channel). The smallest possible scanning area (12.11 × 12.11 μm) with 512 × 512 pixel spatial resolution and 10 Hz scanning speed was chosen for the microcapsules’ photoconversion procedure. Angularly averaged fluorescence intensity of capsules before and after photoconversion was measured using the Gwyddion software 2.63.

### 3.5. Isolation and Cultivation of hMSCs

hMSCs were obtained from the bone marrow of healthy donors who provided signed informed consent. The cells were isolated using a direct plating procedure [58]. Briefly, heparinized whole bone marrow was re-suspended in alpha-MEM (Lonza, Basel, Switzerland) supplemented with 100 IU/mL penicillin, 0.1 mg/mL streptomycin (Biolot, Saint Petersburg, Russia), 10% FBS (Hyclone, South Logan, UT, USA) and 2 mM UltraGlutamin I (Lonza, Basel, Switzerland). After attachment of the cells to the culture plastic, the medium containing non-adherent cells was replaced with fresh culture medium. The culture medium was changed every 3 days until confluence was reached (80%). For passaging, hMSCs were detached using trypsin/EDTA solution (Invitrogen, Carlsbad, CA, USA) for 5 min and replated at a density of 5.0 × 10^3^ cells/cm^2^. Cultures from 2nd–3rd passage were used for further experiments.

### 3.6. Viability Investigation (MTT Test)

hMSCs were plated in a 96-well plate at a density of 5 × 10^3^ cells per well and incubated overnight (5% CO_2_ at 37 °C). Then, 0, 1, 2.5, 5, 10, 25, 50, and 100 thermally treated photoconvertible polymeric microcapsules per cell were added to the wells and incubated for 24 and 48 h. The culture medium was then replaced with 100 μL of fresh medium containing 10% MTT stock solution. The plates were incubated for 3 h at 37 °C. The cell culture without the addition of polymeric capsules was used as a positive control. After incubation, the culture medium with the MTT reagent was carefully removed, and 100 μL of DMSO solution was added to each well and incubated for 15 min to dissolve the formazan crystals. Finally, the absorbance of each well was measured at 570 nm using a spectrophotometer.

### 3.7. Microcapsules Internalization

hMSCs were plated in a 24-well plate at a density of 3 × 10^4^ cells per well and incubated for 24 h under identical conditions. The thermally treated microcapsules were then added to the cells at the ratio of 0, 2.5, 5, 10, and 25 capsules per cell and incubated for 24 h to allow for their internalization. Next, the culture medium was removed, and the cells were washed thrice with PBS to remove uncaptured capsules. For cell staining, 150 μL stock solution with Calcein AM and Hoechst 33258 (12,000 times diluted) in serum-free medium was added to each well and incubated for 30 min. After staining, the growth medium with the dyes was removed, and the cells were washed with PBS and harvested using 0.05% trypsin with EDTA solution. The cells were then supplemented with 2% FBS and measured using an ImageStream X Mark II Imaging flow cytometer (Luminex, USA). The measurement was performed using INSPIRE software (https://inspiresoftware.com/ (accessed on 28 August 2023)) with the following equipment settings: 40× magnification; low flow rate/high sensitivity; 405 nm with a laser power of 10 nW; and 488 nm laser at 5 mW. Internalization assays for each cell line were performed in triplicate for each concentration of added capsules (2.5, 5, 10, and 25 capsules per cell). Each replicate involved the analysis of 3500 objects. The number of internalized capsules was determined using the Spot Count feature of the IDEAS software IDEAS 6.2 (Luminex, USA).

### 3.8. Stability Evaluation of the hMSCs’ Immunophenotype

The stability of the hMSCs’ immunophenotype was evaluated by flow cytometry. For these experiments, the cells were first washed with phosphate-buffered saline (PBS) and incubated with a non-specific blocking buffer containing 1% bovine serum albumin for 30 min. After centrifugation and removal of the blocking buffer, samples were treated with fluorescently conjugated mouse anti-human antibodies for 45 min. The expression of 5 surface markers was analyzed. The following antibodies were used: CD45 FITC (2D1), BD Biosciences; CD105 PE (TEA3/17.1.1), Beckman coulter; CD34 PerCP-Vio700 (AC136), MIltenyi Biotec; and CD90 APC (5E10), BioLegend. Vital dye -.DRAQ-7, BioLegend. Among the surface antigens detected, CD34 and CD45 are hematopoietic stem cell markers and thus are not expected to be expressed by MSCs while the others are MSC-specific markers [46,47]. The stained cells were resuspended using PBS and analyzed in a BD FACSCanto™ Clinical Flow Cytometry System (BD Biosciences, San Jose, CA, USA).

### 3.9. hMSCs Tracking

The hMSCs were cultivated on 35 mm petri dishes (Ibidi GmbH, Martinsried, Germany) at a density of 3 x 10^4^ cells per dish. The cells were stained with a 0.1% solution of cyanine 7 NHS ester in ethanol (6 μL of the solution was added to 200 μL of the medium for 30 min). This ensured stable hMSCs staining and clear visualization in the fluorescent mode of the confocal microscope for 7 days. Thermally treated photoconvertible microcapsules were added 24h before the experiment at a ratio of 10 capsules per cell. Fluorescent images of hMSCs were obtained before and 15 min and 1, 3, 5, and 7 d after microcapsule photoconversion using the confocal microscope. The settings for obtaining the images and photoconversion of the microcapsules were similar to those described above. The cells were visualized using a 671 nm laser with a detection range of 685–790 nm.

## 4. Conclusions

The choice of the labeling method for cell tracing should be largely based on the characteristics of the experiment, the type of cells, and the envisaged plans for the use of the studied population. For a long time, fluorescent photoconvertible proteins have been at the forefront of cell labeling and tracking despite the need for prior gene modification. In addition to the difficulties of transfection of individual lines (especially primary ones, such as hMSCs), the question of using the cultures studied in experiments in personalized medicine is still open.

Here we reported a prospective approach for labeling and tracking individual hMSCs without genetic modification. Photoconvertible polymeric microcapsules have been suggested as labels that can be internalized and retained inside hMSCs for at least 7 days. The efficiency of capsule internalization is directly related to the added capsule concentration. A ratio of 10 capsules per cell is an optimal, causing 46 ± 2% of all cells to be labeled. The consequent increase in the number of capsules led to a critical decrease in cell viability and a change in morphology. At the same time, a higher ratio of capsules to cells does not have a significant effect on the cell immunophenotype. The developed approach made it possible to track the trajectory of the movement of a number of individual cells for several days, as well as the destiny of daughter cells after doubling, without additional marking for 4 days. Despite the fact that the converted and non-converted capsules were split between the daughter cells randomly, the brightness of the label is preserved, and the new color code can be automatically “assigned” to the daughter cells. These new codes can be traced during subsequent monitoring. Reliable tracing of hMSCs and their daughter cells was performed for 1.5 or 2 doubling periods before the culture reached 100% confluence (with an initial confluence value of 25–30%). Nevertheless, it is possible to start the experiment with lower confluence values (10–15%) for some cell cultures that are less sensitive to their microenvironment than hMSCs. In this case, the multiple doublings will eventually lead to the appearance of daughter cells with no capsules or too few capsules for to assign them a “color code”. The repeated addition of microcapsules for labeling can help solve this problem. Note that the current approach using photoconvertible proteins has no possibility of individual cell coding. The fluorescent signal starts to dilute within the first few hours after protein photoconversion due to the continuous synthesis of new unconverted molecules. Splitting of the converted protein during cell doubling also decrease a detectable signal until complete disappearance after 48–72 h [31].

The developed photoconvertible microcapsules have broader prospects for creating stable purified lines because of their ability to detect each cell from the labeled group as well as the absence of gene modification. There is a need to select and isolate specific cell populations from heterogeneous cultures [59]. For example, after labeling, morphologically competent cells can be sorted and clones with the requested characteristics may be obtained. Cells that are capable of rapid doubling or directional movement can be selected and expanded for further use in regenerative medicine.

Generally, the proposed approach proved to have high potential for studying primary cell lines via multiple single-cell tracking. This is especially important for stromal cells including tumor cells, as they are widely differentiable. The developed strategy could become key to understanding their fate in the long-term cell-to-cell cultivation of the whole population.

## Data Availability

The data supporting the findings of this study are available from the corresponding author upon reasonable request.
